# Tumor to Tumor Metastasis from Adenocarcinoma Not Otherwise Specified of the Parotid Gland to Uterine Leiomyoma: Presentation of a Unique Case

**DOI:** 10.7759/cureus.6789

**Published:** 2020-01-27

**Authors:** Alexandros Psarris, Nektarios Koufopoulos, Anastasios Grivas, Dimitrios C Papatheodorou, Lubna Khaldi

**Affiliations:** 1 Gynecology, Anti-Cancer Hospital of “Saint Savvas”, Athens, GRC; 2 Pathology, Attikon University Hospital, Medical School of Athens, Athens, GRC; 3 Oncology, Anti-Cancer Hospital of “Saint Savvas”, Athens, GRC; 4 Pathology, Saint Savvas Cancer Hospital, Athens, GRC

**Keywords:** adenocarcinoma not otherwise specified, parotid gland, uterine leiomyoma, tumor to tumor metastasis, distant metastasis.

## Abstract

Salivary gland adenocarcinoma not otherwise specified (NOS) is a malignant epithelial tumor composed of ductal/glandular structures with or without cystic formation. Histologically it is classified as high grade with relevant biological behavior. Although both minor and major glands may be involved, the majority (60%) implicate the parotid gland. Location, regional lymph node status, and histological grade are some of the factors that predict the progress of the disease and the development of metastases. Long follow-up is considered the standard option as distant metastases (DM) may occur despite regional control. Primary sites of DM, besides lymph nodes, include bone, lung, and liver. Herein we report a unique case of a 68-year-old female with a previous history of high-grade adenocarcinoma NOS of her right parotid gland. On her biannual follow-up examination, MRI revealed an abnormal increase in the size of a known uterine leiomyoma of the posterior uterine wall. Positron emission tomography-CT (PET-CT) showed increased uptake in the uterus and lungs. On frozen section, adenocarcinoma was found at the center of the leiomyoma. Histological and immunohistochemical findings were consistent with secondary involvement by the salivary gland adenocarcinoma NOS. Treatment consisted of cyclophosphamide, adriamycin, and cisplatin with poor outcome. The patient was lost to follow-up. Review of the literature indicates that no similar case has been reported in the English literature.

## Introduction

Salivary gland adenocarcinoma not otherwise specified (NOS) is a highly malignant tumor [1-2], which histologically presents itself with ducts/glands without specific morphological and/or immunohistochemical features. It is characterized as a diagnosis by exclusion [3]. Higher grade tumors may show solid nests and pleomorphism. Both major and minor salivary glands are involved with a predilection to the parotid gland. Tumors of minor salivary glands have a better prognosis than those of major salivary glands [3]. Distant metastases (DM) may occur despite regional control and are most commonly found in the lungs, bones, and brain. However, several unusual sites of metastasis have been described in the literature, such as the stomach, thyroid, skeletal muscles, and skin.

Uterine leiomyomas are very common benign neoplasms composed mainly of smooth muscle tissue. Metastases to uterine leiomyomas from extragenital tumors are uncommon. In the vast majority of cases, metastases originate from the breast, colon, stomach, pancreas, gallbladder, lung, urinary bladder, thyroid, and melanoma of the skin [4].

The case in our study is unique in presentation. It concerns an adenocarcinoma NOS of the parotid gland with simultaneous metastases to the lungs and uterine leiomyoma. After a thorough review of the literature, we found no similar cases.

## Case presentation

**Figure 1:**
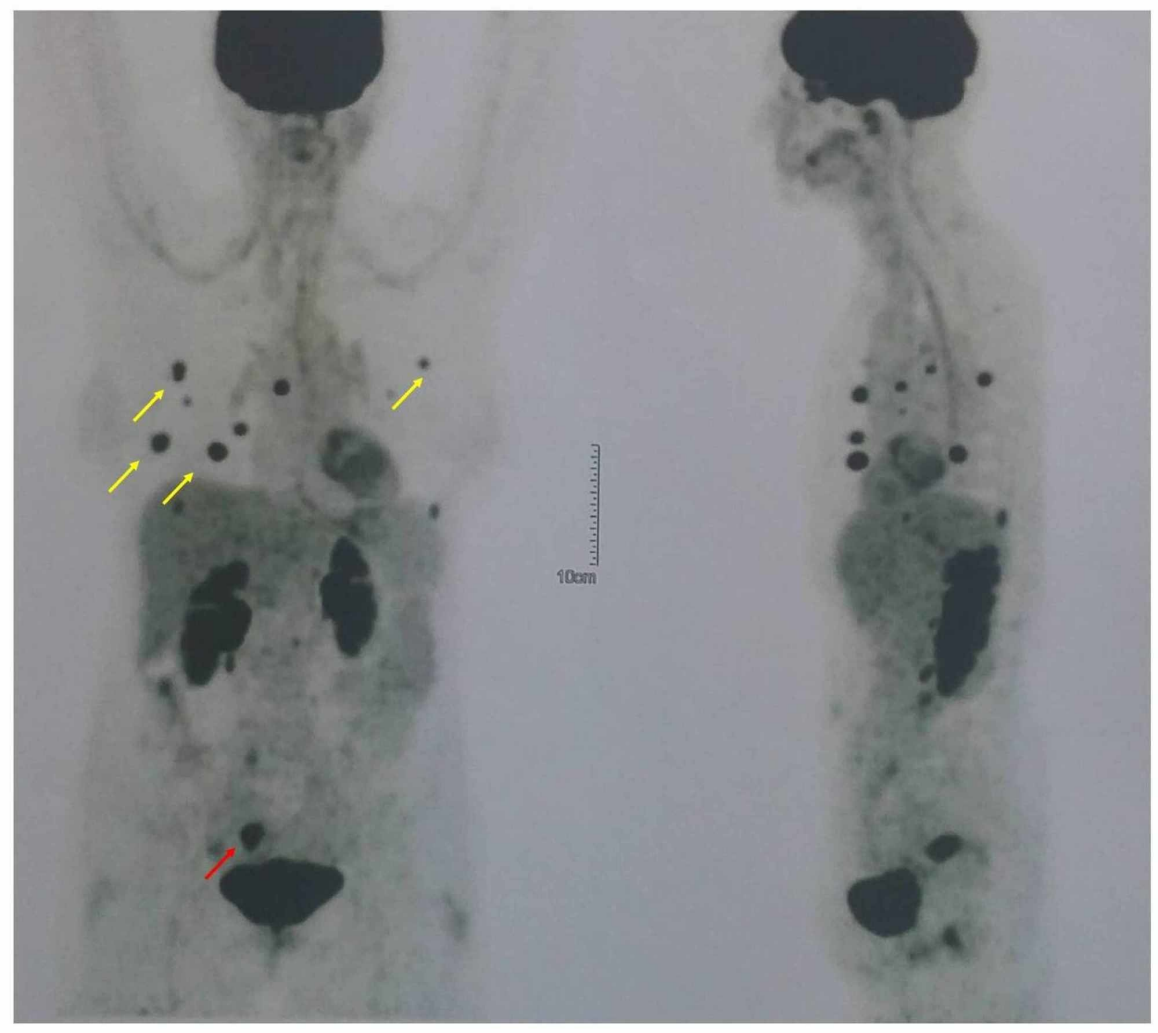
PET-CT scan showing 18F-FDG uptake in the uterus (red arrow) and multiple nodules of both lungs (yellow arrows) PET-CT: positron emission tomography-computed tomography; 18F-FDG: fludeoxyglucose F 18

A 68-year-old female patient was referred to our hospital because of a pelvic mass. The patient’s history included high-grade adenocarcinoma of the salivary gland diagnosed four years earlier when she underwent a right parotid salivary gland resection followed by concurrent chemoradiation with weekly cisplatin 30 mg/m^2^. Past medical history revealed laparoscopic cholecystectomy 10 years ago, and a palpable smooth uterine mass consistent with leiomyoma diagnosed initially 20 years ago. At present, during routine follow-up of her parotid tumor, MRI imaging of the lower abdomen revealed that the known leiomyoma of the posterior uterine wall had increased in size, measuring 7 cm x 5 cm and appeared to have heterogeneous texture. Positron emission tomography-CT (PET-CT) showed increased fludeoxyglucose F 18 (18F-FDG) uptake in the uterus (SUVmax 11.1) and multiple nodules of both lungs (SUVmax 8.9) (Figure 1). Bronchial washings were negative, and CT-guided fine needle aspiration (FNA) showed atypical cells suspicious for malignancy. The patient underwent a total abdominal hysterectomy and bilateral salpingo-oophorectomy.

 

**Figure 2:**
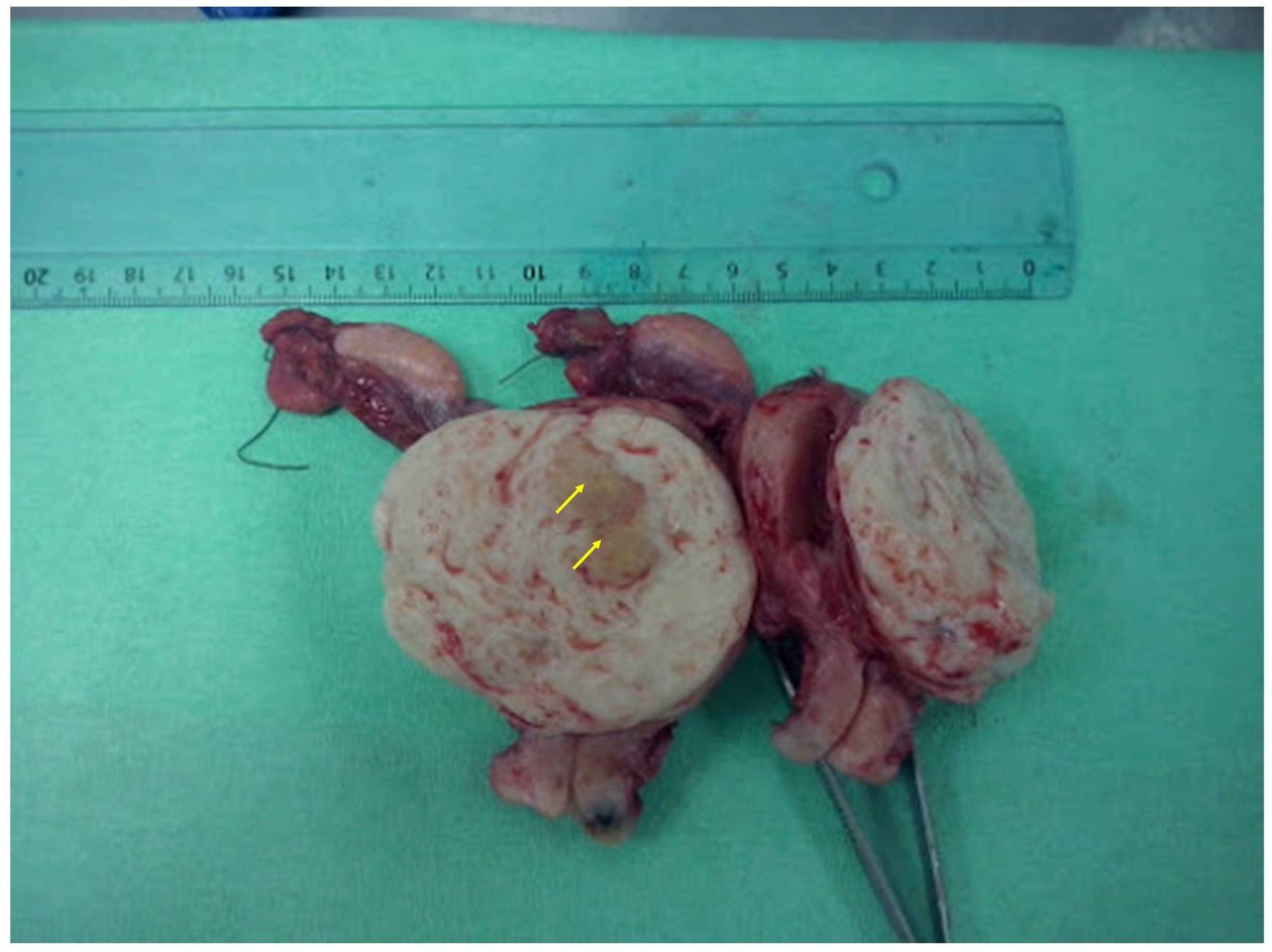
Image of the uterus during frozen section examination. The tumor can be seen at the center of the leiomyoma (yellow arrows).

Grossly, the uterus was enlarged with a large (6 cm) leiomyoma. At its center, a distinct yellowish lesion was noticed measuring 2.8 cm in its greatest diameter (Figure 2). Frozen section required by the surgeon revealed the presence of adenocarcinoma.

**Figure 3:**
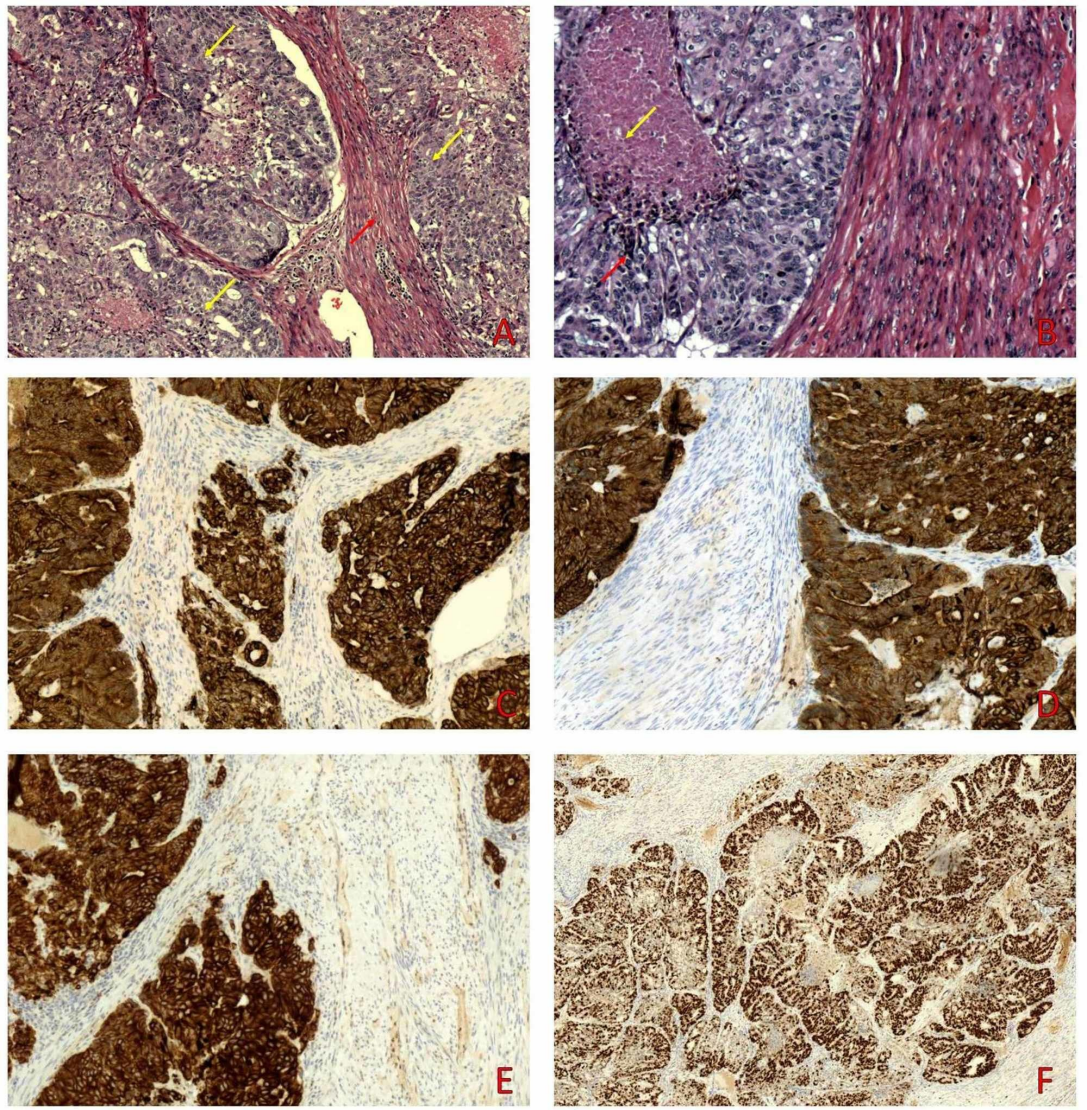
A. On medium power examination the tumor (yellow arrows) infiltrates the leiomyoma (red arrow). B. Some tumor nests (red arrows) display central necrosis (yellow arrows). Immunohistochemical study was positive for AE1/AE3 (C), cytokeratin 8/18 (D), and cytokeratin 7 (E). Ki67 stained more than 90% of tumor nuclei (F).

Permanent hematoxylin and eosin sections depict ductal structures as well as solid nests of variable size surrounded by fibrous connective tissue. The tumor cells showed intermediate grade, limited nuclear pleomorphism, hyperchromatic nuclei, and infrequent mitotic figures (Figure 3A). Foci of necrosis were also present (Figure 3B). Immunohistochemical study showed positivity for AE1/AE3 (Figure 3C), cytokeratin 8/18 (Figure 3D), and cytokeratin 7 (Figure 3E). Immunostains were focally positive for C-erbB-2 and weakly positive for GATA3. Tumor cells failed to express cytokeratin 20, CDX-2, SMA, S-100, HMB45, Vimentin, PR, TTF-1, and Napsin A. Ki67 proliferation index stained more than 90% of tumor cells (Figure 3F).

Pathology report based on immunochemistry excluded metastasis from the lung as well as the possibility of an occult lesion from the breast, colon, thyroid, and melanoma. Gastroscopy revealed no abnormalities in accordance with blind gastric sampling biopsy. Hence, the diagnosis of the known parotid gland adenocarcinoma metastasis to the uterine leiomyoma was established. Recurrent disease was treated according to CAP chemotherapy regimen (cyclophosphamide 600 mg/m^2^, adriamycin 50 mg/m^2^, cisplatin 50 mg/m^2^). After three cycles of chemotherapy, the disease remained stable, and chemotherapy was discontinued due to hematological toxicity despite dose reductions. The patient experienced fast progression, and second-line weekly paclitaxel was administered with poor outcome. She was finally lost to follow-up.

## Discussion

Adenocarcinoma NOS of the salivary gland is a highly malignant tumor [1-2], characterized by ductal formation devoid of specific morphological and/or immunohistochemical features. The frequency of the tumor is indeterminate because some pathologists report NOS as adenocarcinoma only, which applies to most salivary gland malignancies. The tumor involves both major and minor salivary glands in about 60% and 40%, respectively, reported in WHO 2005 edition; those percentages were modified to more than 50% and 40%, respectively, in WHO 2017 edition [5-6]. Parotid gland predilection was recorded in 60% of cases, and the peak age incidence is the sixth decade [3]. The 15-year survival rate is 54% for low-grade tumors and 3% for high grade [3].

Recently a descriptive and retrospective analysis of adenocarcinomas NOS came to light concerning 3155 patients covering a period from 1998 to 2012 and analyzing numerous variables using the National Cancer Database of the United States [7]. The authors emphasized on the fact that data review started in 1992, namely, took place after the significant classification changes. Comparison of epidemiological studies about gender preponderance showed that the overall male to female percentage was 62.8% to 37.2% [7]. Specifically, low-grade tumors percentage was male to female ratio 45.6% to 54.4%, while high-grade tumor percentage was male to female ratio 68.9% to 31.1%. Likewise, Gallo et al. studied patients from 1970 to 1990 and reported a slight female preponderance in low-grade carcinomas (female to male ratio 22:17) and males outnumbering females in high-grade tumors (56:29) [8]. Another observation is that patients with low-grade carcinomas of the parotid gland had a median age of 51 years, irrespectively of tumor type, and 67 years for high-grade tumors [8], while Zhan et al. found a median age of 67 years for adenocarcinomas NOS [7].

Although the first study was conducted before the modification of the classification, and the second one after, the trend in both investigations coincides.

Distant metastases of carcinomas in the head and neck region are, in general scarce. According to the histological type, the overall incidence of distant metastases ranges between 20% and 50%. The mortality rate of metastatic disease was 63.6% [8]. Renehan et al., after analyzing 825 patients, covering a 40-year period (1952-1992), concluded that the incidence of parotid tumors was 24% [9]. A study from the Memorial Sloan-Kettering Cancer Center found that DM appeared in 18.9% of cases [2]. Nam et al. reported a 7.4% incidence of carcinomas of the salivary gland at the initial stage, which increased up to 92.6% during the 100-month follow-up, with multiple organs involvement in 32.6% of those patients [10]. Similarly, for adenocarcinomas NOS Zhan et al. referred to an incidence of 7.9%. More specifically, DM was found in 9.5% of cases [7]. The observed variations in the incidence probably rely on the mixture of different histological tumor types, tumor sites, as well as the duration of follow-up periods.

At present, it is generally accepted that DM may occur despite regional control and regardless of histological type. Namely, Deng et al. found that 14.3% of patients with NOS adenocarcinomas had positive margins on permanent sections despite the negativity of margins on frozen section [11]. Gallo et al. suggested that locoregional control is independent of metastases in patients with parotid tumors [8].

Distant metastases were confined to the lungs, bones, liver, soft tissue, lymph nodes, brain, kidney, orbit, and pancreas [1-2], although reports of individual cases refer odd metastases to the skin, skeletal muscle, even thyroid and stomach. Clinical predictors for DM are male gender (2.1 times more than female), clinical T4, and clinically positive lymph nodes [2].

Histological parameters that predict the risk of DM are tumor size, grade, jugular lymph node involvement, local spread to surrounding soft tissue as well as facial nerve impairment and vascular invasion [1-2, 9]. Particularly, large tumor size was found to correlate significantly with the development of DM [9]. Furthermore, high-grade tumors had twice the metastatic rate of low-grade carcinomas [8]. Recent data raise the possibility of DM up to 7.5 times in cases with high-grade tumors [2]. Additionally, a higher number of regional lymph node involvement implies 3.4 times higher risk of having DM [2]. Perineural invasion is present in 20% of patients and anticipate higher local recurrence rate as well as poor prognosis [12]. Likewise, Ali et al. found that perineural invasion increases the risk of DM by five times [2]. Finally, tumors of minor salivary glands have a better prognosis than that of major [3].

Metastases to uterine leiomyomas are uncommon. They are caused either by direct extension from genital tumors as well as tumors of adjacent organs or by hematogenous spread. The most frequent extragenital tumors that give metastases to leiomyomas-sited by frequency are breast, colon, stomach, pancreas, gallbladder, lung, melanoma of the skin, urinary bladder, and thyroid [4].

Salivary gland carcinomas are rare and include a wide variety of tumor types. Treatment recommendations are based mainly on retrospective reviews and clinical experience. The cornerstone of treatment is surgical resection with negative margins if this is feasible. High-risk patients typically undergo adjuvant radiotherapy [13]. The addition of chemotherapy has not proven efficacy based on retrospective data [14]. Metastatic disease has variable biologic behavior depending on histologic subtype. The main goal of medical approach in metastatic disease is palliation and should be tailored to biologic behavior of the disease. Watchful waiting is a rational option for indolent disease, and chemotherapy regimens containing cisplatin, doxorubicin, and cyclophosphamide may be reserved for symptomatic disease [15]. There is also some evidence that paclitaxel is active in adenocarcinomas but not in adenoid cystic carcinomas [16]. Targeted therapy has achieved some objective and durable responses in early-phase clinical trials and refers to HER-2 inhibition and multiple tyrosine kinase inhibitors. Antiandrogen hormone therapy has shown some activity in those tumors which express the androgen receptor, and checkpoint immunotherapy is being tested in ongoing clinical trials. Oligometastatic disease resection may be considered in highly selected patients.

## Conclusions

In summary, we present a case of adenocarcinoma NOS with metastases to the lungs and uterine leiomyoma. Metastases to uterine leiomyomas are very rare. Our case is unique as it is the first report of a salivary gland carcinoma metastasizing to a leiomyoma. The fact that no similar case has been reported advocate circumstance therapy. There is no clear evidence that survival is prolonged by systemic therapy or total parotidectomy with facial nerve sacrificing. The role of molecular targeted therapy has not been well established yet. Follow-up of carcinomas of the parotid gland should be lifelong.
